# A phase II study of intraoperative radiotherapy using a low-energy x-ray source for resectable pancreatic cancer: a study protocol

**DOI:** 10.1186/s12893-019-0492-x

**Published:** 2019-03-07

**Authors:** Jun Won Kim, Yeona Cho, Hyung Sun Kim, Won Hoon Choi, Joon Seong Park, Ik Jae Lee

**Affiliations:** 1 Department of Radiation Oncology, Gangnam Severance Hospital, Yonsei University College of Medicine, 211 Eonju-ro, Gangnam-gu, Seoul, 06273 Republic of Korea; 2 Department of Pancreatobiliary Surgery, Gangnam Severance Hospital, Yonsei University College of Medicine, 211 Eonju-ro, Gangnam-gu, Seoul, 06273 Republic of Korea

**Keywords:** Resectable pancreatic cancer, Intraoperative radiotherapy, Low-energy x-ray source, Local control

## Abstract

**Background:**

The current standard treatment for resectable pancreatic cancer is surgical resection followed by adjuvant chemotherapy. Local recurrence rates are high even after curative resection; thus, the long-term outcome of locally advanced pancreatic cancer remains poor. Intraoperative radiotherapy (IORT) uses a low-energy x-ray source to deliver a single fraction of high-dose radiation to the tumor bed during a surgical procedure, while effectively sparing the surrounding normal tissues. IORT has the potential to improve the efficacy of radiation therapy for pancreatic cancer.

**Methods/design:**

This prospective, one-armed, phase II study will investigate the role of IORT in improving local control in patients with resectable pancreatic adenocarcinoma. The patients will receive surgery and IORT of 10 Gy prescribed at a 5-mm depth of the tumor bed, followed by adjuvant gemcitabine chemotherapy according to the current standard of care. The aim is to enroll 42 patients.

**Discussion:**

The primary endpoint of this trial is to evaluate the feasibility of IORT and the local recurrence rate after one year. The secondary endpoints include the acute and late toxicities, and disease-free survival and overall survival rates.

**Trial registration:**

The trial was prospectively registered at Clinicaltrials.gov NCT03273374 on September 6, 2017.

## Background

Pancreatic cancer is the fourth most common cause of cancer-related mortality in the Western world [1, 2]. Although, surgical resection offers potentially curative treatment for pancreatic adenocarcinoma, only 15–20% of patients are candidates for curative pancreatectomy [3]. Even after successful surgical resection, long-term survival is rare, and the five-year survival rate of patients with resected pancreatic adenocarcinoma is approximately 10% [4]. The predominant sites of recurrences are both local and distant in the majority of patients who undergo pancreatic resection, suggesting that most patients may have occult metastatic or local/regional disease (or both) at the time of surgery. Several prospective studies have shown that combined modality approaches using adjuvant chemotherapy or chemoradiation in addition to surgery can improve locoregional control and survival [5–7].

Previous trials involving external beam radiation therapy (EBRT) have shown improved local control in combination with surgical resection [6, 8]. However, the efficacy of EBRT in pancreatic cancer is limited by the difficulty of delivering an adequate dose of radiation due to the limited tolerance of critical organs, including the stomach, small bowel, kidney, liver, and spinal cord. Furthermore, adjuvant EBRT concurrently administered with gemcitabine is excluded from reimbursement by the Korean National Health Insurance. During a surgical procedure, intraoperative radiation therapy (IORT) delivers a single fraction of high-dose radiation to the tumor bed after gross total resection or remaining residual tumor. IORT has the potential to improve the efficacy of radiation therapy for pancreatic cancer by reducing radiation dose to the normal tissue and allowing the escalation of radiation dose to the tumor bed to further improve local control [9, 10]. This is important in pancreatic cancer because local failure rates are as high as 50–80% in patients with resected and locally advanced disease [11].

In a conventional IORT procedure, the patient has to be transferred from the operating theater to a shielded radiation treatment room during open laparotomy or the entire operation must be performed in a radiation treatment room which is inadequately equipped for surgical operation [12]. Intrabeam (Carl Zeiss, Germany) is a mobile IORT system equipped with a miniature x-ray source of 50 kV that allows an entire IORT procedure to be performed inside an operating theater, eliminating the need for patient transfer during high-risk operations such as pancreatoduodenectomy [13]. The low-energy, 50 kV peak x-ray has dose attenuation characteristics that follow the inverse cubic law (1/r^3^). The steep dose gradient generated by the Intrabeam system allows the delivery of high-dose radiation to the tumor bed surface while effectively limiting delivery of significant doses to the surrounding normal tissues [14]. Furthermore, the relative biologic effectiveness (RBE) of 50-kV x-rays from the Intrabeam at depths clinically relevant for tumor-bed irradiation was determined to be 1.26 to 1.42 [15], thus increasing the likelihood of successful local control in patients undergoing IORT using the 50-kV x-ray source.

## Methods/design

### Hypothesis

This trial tests the hypothesis that IORT in addition to the current standard of care will improve local control compared with that observed so far in patients with resectable pancreatic adenocarcinoma.

### Study objectives and endpoints

The primary endpoint is to evaluate the local recurrence rate after one year. The secondary endpoints include disease-free survival (DFS) and overall survival (OS) rates as well as acute and late toxicities, perioperative morbidity and mortality, and quality of life.

### Study design and period

The purpose of the study is to investigate the role of IORT in patients with primarily resectable pancreatic cancer. The trial will be conducted as a single-center, one-armed phase II study. The trial has been registered at www.clinicaltrials.gov (NCT03273374). Patients will be recruited at the Pancreatobiliary Cancer Center, Gangnam Severance Hospital, Yonsei University College of Medicine. The expected total duration of patient enrollment is two years and the follow-up period is three years.

### Patient selection and enrollment criteria

#### Inclusion criteria

- 20 years of age or older
- Histologically confirmed adenocarcinoma of the pancreas
- Eastern Cooperative Oncology Group performance status scores of 0–2
- Resectable disease defined as follows:
  - Absence of distant metastases
  - Clear fat planes around the celiac axis, hepatic artery, and superior mesenteric artery
  - Absence of direct involvement of inferior vena cava or aorta
- Stage I–III disease per the seventh edition of the American Joint Committee on Cancer
- Adequate bone marrow function (hemoglobin > 10 g/dL, absolute neutrophil count > 1500/mm^3^, platelets > 100,000/mm^3^)
- Adequate renal function (serum creatinine < 1.4 mg/dL, BUN < 20 mg/dL)
- Written informed consent

#### Exclusion criteria

- Prior EBRT in the abdominal area
- A tumor bed which cannot be adequately covered by the IORT field as defined by the radiation oncologist
- Neoadjuvant chemotherapy
- Unresectable disease
- Presence of distant metastases
- Pregnancy or currently nursing

### Sample size calculation

The primary endpoint of the trial is the local recurrence rate after one year. The study is designed to demonstrate that IORT using a low-energy x-ray source delivered to the tumor bed of the resected pancreatic cancer followed by standard adjuvant chemotherapy can improve the local recurrence rate after one year. The local recurrence rate one year after resection in our institution was 36%; the local recurrence rates after one year in comparable patient populations treated with IORT and resection ranged between 21 and 41.6% [16–19]. The sample size calculation was designed on the assumption that addition of IORT will decrease the local recurrence rate after one year by 14% (from 36 to 22%) with a power of 80%. Using the two-sided binomial test with a level of significance of α = 5%, the study requires 33 patients. Assuming a drop-out rate of 20%, a total of 42 patients will be required for this trial.

### Pretreatment evaluation

The initial workup will consist of physical examination, laboratory tests including tumor markers, computed tomography (CT) or magnetic resonance imaging (MRI) of the abdominal cavity, thoracic CT, and biopsy. Positron emission tomography (PET) will be performed, when necessary, to detect distant metastasis. Percutaneous or endoscopic biliary drainage will be recommended for patients with obstructive jaundice before or during treatment.

### Treatment

**Fig. 1:**
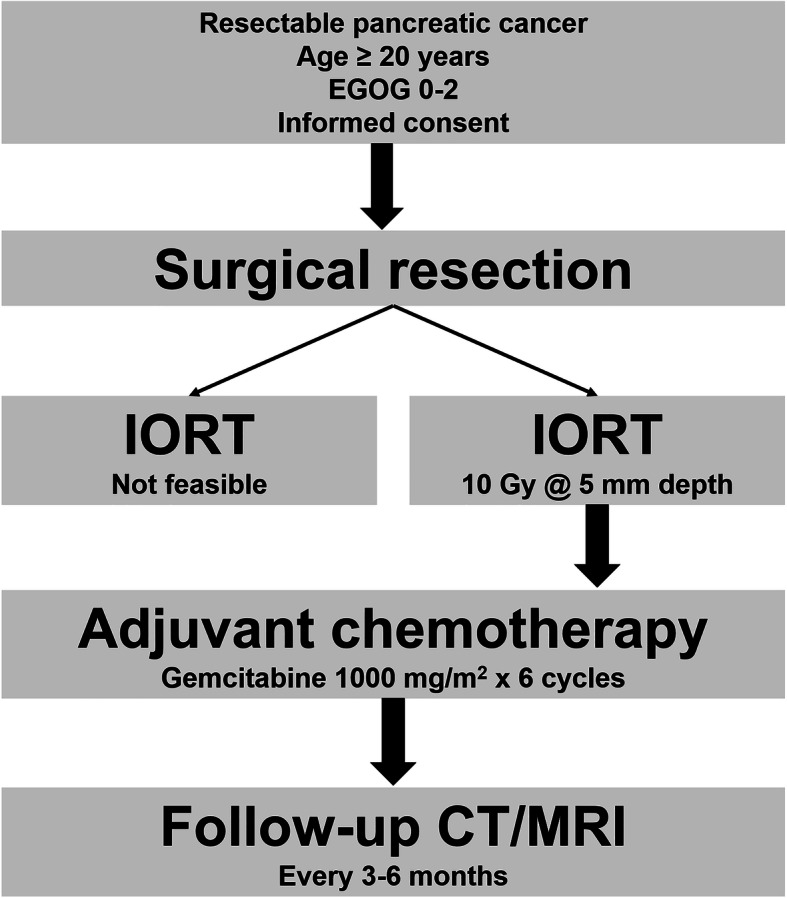
Treatment schedule

All patients who fulfill the inclusion criteria and provide written informed consent will be assigned to the treatment regimen shown in Fig. 1. An explorative laparotomy will be performed and the indication to continue with a resection will be based upon the absence of peritoneal or distant metastasis and the loco-regional extension of the disease, particularly major vascular involvement. A curative resection either as pancreatoduodenectomy or distal pancreatectomy will be performed.

**Fig. 2:**
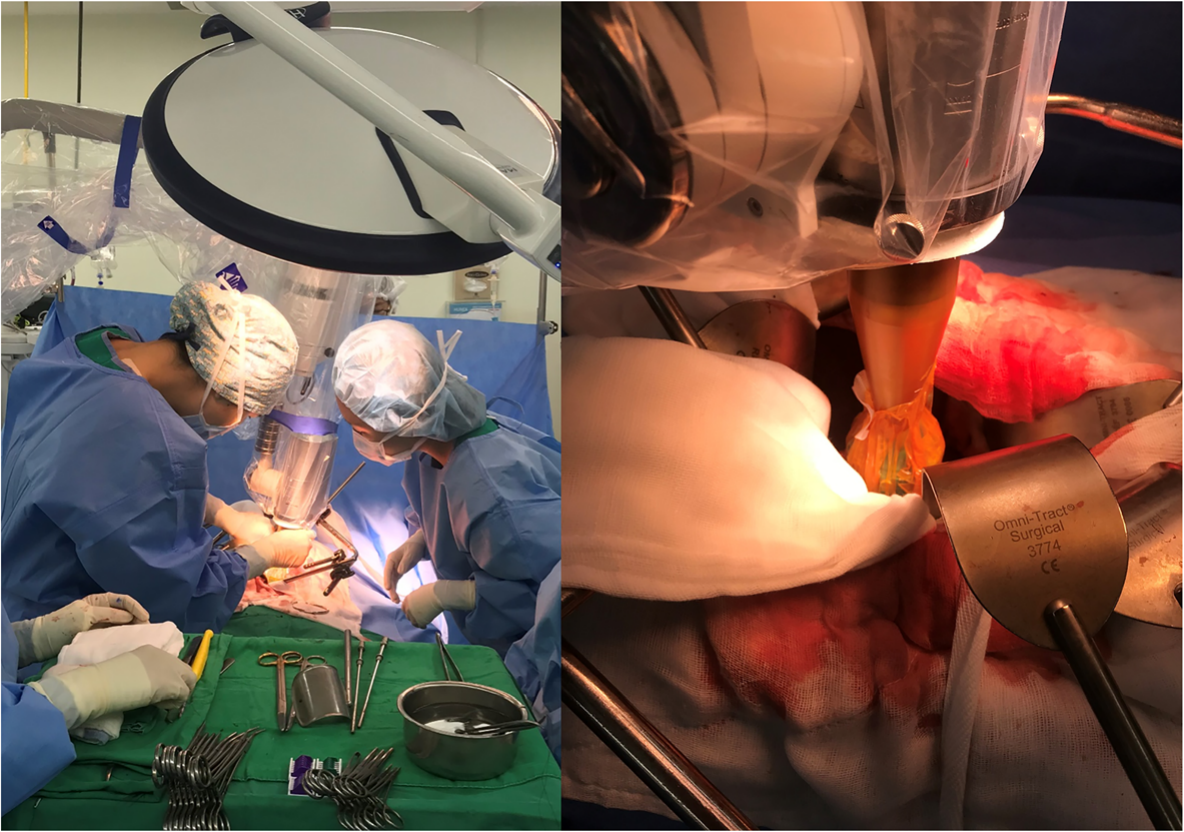
The Intrabeam IORT procedure

The mobile 50-kV x-ray source (Intrabeam) will be attached to the robotic arm, which maintains the stability of the source throughout the procedure (Fig. 2). The isotropy and output of the IORT unit are verified and the pre-IORT calibration process required by the system is performed prior to each treatment. The target volume of IORT includes the tumor bed, the celiac and superior mesenteric origins, the mesenteric root, and the portal vein or any areas deemed risky by the surgeon and the radiation oncologist. An applicator with an appropriate diameter (3.0, 3.5, or 4.0 cm) will be selected according to the size of the target volume, and the applicator attached over the probe of the x-ray source. A sterile sheath will be draped over the IORT device to prevent contamination, and the applicator will be placed on the tumor bed by a radiation oncologist in correspondence with the treating surgeon. Uninvolved radiosensitive tissues will be removed and shielded from the treatment area. After locking the device in a treatment position and placing additional shielding over the surgical field to protect the operators, the target volume will be irradiated with a single dose of 10 Gy, prescribed at a 5-mm depth into the tumor bed. Eight to 12 weeks following surgery or after wound healing, the patients will receive adjuvant chemotherapy with six cycles of gemcitabine every four weeks. Each chemotherapy cycle consists of three weekly gemcitabine doses of 1000 mg/m^2^ administered by intravenous infusion during a 30-min period, followed by one week of rest [20].

### Follow-up and assessment of efficacy

Regular follow-up visits will take place every three months after surgery for the first two years and every six months thereafter. Patients will be followed with physical examinations, tumor markers measurement, imaging studies including CT or MRI of the abdominal cavity, and PET-CT when necessary.

The local recurrence rate after one year is the primary endpoint of the trial. It will be assessed by repeated CT, MRI, or PET-CT during regular follow-up. In case of suspected local recurrence, histological confirmation will be attempted. New lesions with typical radiological signs of a local recurrence in combination with or without increase in the levels of tumor markers will be considered a local recurrence. DFS and OS are the secondary endpoints of the study. DFS will be counted from the day of surgery until the date of the first local or distant event or death due to any cause. Patients alive without recurrent disease at the time of data analysis will be censored at the time of the most recent follow-up. The OS will be determined from the day of surgery until death due to any cause. For timed endpoints including DFS and OS, the Kaplan-Meier survival analysis followed by multivariable Cox proportional hazards model for adjusting for baseline variables will be used. *P*-values < 0.05 will be considered significant. Patients alive or lost to follow-up will be censored at the date of the last follow-up visit. Toxicity will be assessed according to Common Terminology Criteria for Adverse Events Version 3.0. Any toxicity occurring within three months after surgery will be considered acute toxicity. Late toxicity will be assessed during the regular follow-up visits.

### Safe evaluation and reporting of adverse effects

Adverse and serious adverse events must be reported in order to protect participants. Study participation will be terminated, if the patient suffers from a grade 4 toxicity related to treatment, if a different treatment is required that is not approved in this trial, or if the patient withdraws consent for further participation. Serious adverse events will be reported within seven days of detection by the investigators.

## Discussion

This trial investigates whether the addition of IORT using a low-energy x-ray source to the standard treatment, consisting of surgical resection (pancreatoduodenectomy or distal pancreatectomy) followed by adjuvant chemotherapy, improves the local recurrence rate after one year. The treatment outcomes of pancreatic cancer remain poor and local failure rates are as high as 50–80% in patients with resected and locally advanced disease [11]. The physical characteristics and relative biological effectiveness of 50 kV x-ray allow high-dose irradiation of the tumor bed while effectively sparing the surrounding normal tissues [14, 15]. The mobile Intrabeam IORT system also allows a single-fraction treatment during surgery without having to transfer the patient out of the operating theater.

The treatment schedule in this study consists of pancreatoduodenectomy or distal pancreatectomy and IORT followed by adjuvant chemotherapy of full-dose gemcitabine (1000 mg/m^2^). Unlike adjuvant EBRT, IORT does not require delayed administration or dose reduction of adjuvant gemcitabine due to the increased risk of radiation toxicity with concurrent administration. Furthermore, IORT allows an additional treatment option of adjuvant EBRT for high-risk patients such as those with narrow R0 or R1 resection in the final pathology report.

IORT with a mobile low-energy x-ray source has potential to improve local control; this phase II trial will evaluate the role of IORT in patients with resectable pancreatic cancer.

## Abbreviations

CT: Computed tomography
DFS: Disease-free survival
EBRT: External beam radiation therapy
IORT: Intraoperative radiotherapy
MRI: Magnetic resonance imaging
OS: Overall survival
PET: Positron emission tomography
